# Evaluation of a Typhoid/Paratyphoid Diagnostic Assay (TPTest) Detecting Anti-*Salmonella* IgA in Secretions of Peripheral Blood Lymphocytes in Patients in Dhaka, Bangladesh

**DOI:** 10.1371/journal.pntd.0002316

**Published:** 2013-07-11

**Authors:** Farhana Khanam, Alaullah Sheikh, Md. Abu Sayeed, Md. Saruar Bhuiyan, Feroza Kaneez Choudhury, Umme Salma, Shahnaz Pervin, Tania Sultana, Dilruba Ahmed, Doli Goswami, Md. Lokman Hossain, K. Z. Mamun, Richelle C. Charles, W. Abdullah Brooks, Stephen B. Calderwood, Alejandro Cravioto, Edward T. Ryan, Firdausi Qadri

**Affiliations:** 1 International Centre for Diarrhoeal Disease Research (icddr,b), Bangladesh, Dhaka, Bangladesh; 2 Department of Microbiology, Dhaka Medical College and Hospital (DMCH), Dhaka, Bangladesh; 3 Division of Infectious Disease, Massachusetts General Hospital, Boston, Massachusetts, United States of America; 4 Department of Medicine, Harvard Medical School, Boston, Massachusetts, United States of America; 5 Department of Microbiology and Immunobiology, Harvard Medical School, Boston, Massachusetts, United States of America; 6 Department of Immunology and Infectious Diseases, Harvard School of Public Health, Boston, Massachusetts, United States of America; 7 International Vaccine Institute, Seoul, South Korea; Oxford University Clinical Research Unit, Viet Nam

## Abstract

**Background:**

Rapid and reliable diagnostic assays for enteric (typhoid and paratyphoid) fever are urgently needed. We report the characterization of novel approach utilizing lymphocyte secretions, for diagnosing patients with enteric fever by the TPTest procedure.

**Methodology:**

TPTest detects *Salmonella*-specific IgA responses in lymphocyte culture supernatant. We utilized TPTest in patients with suspected enteric fever, patients with other illnesses, and healthy controls. We also evaluated simplified modifications of TPTest for adaptation in laboratories with limited facilities and equipment.

**Principal Findings:**

TPTest was positive in 39 (27 typhoid and 12 paratyphoid A) patients confirmed by blood culture and was negative in 74 healthy individuals. Among 32 individuals with other illnesses, 29 were negative by TPTest. Of 204 individuals with suspected enteric fever who were negative by blood culture, 44 were positive by TPTest and the patients were clinically indistinguishable from patients with confirmed bacteremia, except they were more likely to be under 5 years of age. We evaluated simplifications in TPTest, including showing that lymphocytes could be recovered using lysis buffer or buffy coat method as opposed to centrifugation, that incubation of cells at 37°C did not require supplemental CO_2_, and that results were available for majority of samples within 24 hours. Positive results by TPTest are transient and revert to negative during convalescence, supporting use of the test in endemic areas. The results can also be read using immunodot blot approach as opposed to ELISA. Since no true gold standard currently exists, we used a number of definitions of true positives and negatives. TPTest had sensitivity of 100% compared to blood culture, and specificity that ranged from 78–97% (73–100, 95% CI), depending on definition of true negative.

**Conclusion:**

The TPTest is useful for identification of patients with enteric fever in an endemic area, and additional development of simplified TPTest is warranted.

## Author Summary

*Salmonella enterica* serotype Typhi and Paratyphi A are responsible for typhoid and paratyphoid fever respectively and the disease caused is known generally as enteric fever. Appropriate and early diagnosis of the disease is important for initiation of treatment of the patient with a suitable antibiotic. The performance of the available diagnostic methods take time and as well as have low sensitivity and specificity. We describe here an immunodiagnostic assay, the TPTest, which is based on the use of secretions of antibodies from peripheral blood lymphocytes. We describe simplifications of the procedure and show that the assay has as a sensitivity of 100% with a specificity that ranges from 78–97% (73–100, 95% CI) for detecting patients with typhoid and paratyphoid fever in an enteric fever endemic zone. The TPTest uses a small blood volume, and reverts to negative by convalescence, supporting its further development as a relatively low cost assay to diagnose patients with enteric fever in endemic zones.

## Introduction

*Salmonella enterica* serotype Typhi and Paratyphi A and B are human-restricted pathogens and are the causes of enteric fever. *Salmonella enterica* serotype Typhi (*S.* Typhi) infection causes approximately 20 million cases of enteric (typhoid) fever and over 200,000 deaths annually [1]. *Salmonella enterica* serotype Paratyphi A (*S.* Paratyphi A) infection causes an additional 5 million cases of enteric (paratyphoid) fever each year [1], [2]. *S.* Paratyphi B are less common causes of enteric fever. The manifestations of enteric fever range from non-specific febrile illness, to prolonged illness characterized by high fever, lymphadenopathy, hepatosplenomegaly, encephalopathy, and complications due to necrosis of ileocecal lymphoid tissue [3]–[5]. Although paratyphoid fever was considered a less severe form of enteric fever, recent longitudinal studies suggest that clinical manifestations of enteric fever caused by *S.* Typhi and *S.* Paratyphi A are similar [2], [6].

In developing countries where typhoid fever is endemic, the incidence ranges from 25 to 1000 cases/100,000 person-years [1], [7], [8]. In highly endemic areas, typhoid fever most commonly occurs among children 1–5 years of age [4], [8]–[10]. For instance, in a fever surveillance study in an urban area in Kamalapur in Dhaka, Bangladesh, the incidence of *S.* Typhi bacteremia for all age groups was 390 episodes/100,000 person-years [10]. The incidence of *S.* Typhi bacteremia among individuals >5 years of age was 210 episodes/100,000 person years, and among children <5 years of age, 1870 episodes/100,000 person-years. Thus, children <5 years of age had an 8.9-fold increased risk of infection when compared with all others (95% CI: 4.9–16.4) [10]. Historical mortality rates of enteric fever are in excess of 15%, but outcomes are improved in the setting of appropriate antibiotics and supportive care [11]–[13]. The management of patients with enteric fever is confounded by the non-specific clinical presentation, inadequate current diagnostic tests, and widespread antibiotic resistance.

The most commonly used diagnostic assay for typhoid fever globally is the Widal test, developed over a century ago, but the Widal assay is neither sensitive nor specific for diagnosing patients with typhoid fever [14]–[16]. Blood cultures are not routinely used in resource-poor countries, and are only 30–70% sensitive [1], [17], [18]. Other assays that have been evaluated include nucleotide amplification assays, and a number of antibody-based detection systems including ELISA, dot blot immunoassay, hemagglutination, coagglutination, and counter immune electrophoresis. Many of these assays lack sensitivity and/or specificity in areas of the world endemic for enteric fever and *Salmonella* infections [19]–[21]. For instance, commercially available rapid tests for typhoid, including Typhidot, Tubex, latex agglutination assay, immuno-chromatographic lateral flow assays and dip stick assays have low utility in identifying patients with enteric fever at the acute stage of disease in endemic zones, when the ability to make a diagnosis is most important [22]–[24]. The sensitivity and specificity of conventional diagnostic methods (blood culture and Widal test) for paratyphoid fever is also comparably low [25]–[27]. A reliable diagnostic method that could be used during the acute phase of both typhoid and paratyphoid fever is therefore needed.

We have previously shown that we could detect IgA antibodies targeting *Salmonella enterica* serotype Typhi in the blood of patients with typhoid fever in Bangladesh, using a lymphocyte culture supernatant (ALS)-based system that targeted serotype Typhi membrane preparation (MP) as target antigen. This assay takes advantage of the transient systemic migration of activated lymphocytes after infection, allowing recovery of antigen-specific lymphocytes in the peripheral circulation. Using this assay, we showed that all (100%) blood culture confirmed typhoid fever patients were detected by this technique [28]. Since this method requires the ex-vivo culturing of isolated lymphocytes and ELISA, we focused our efforts on simplifying the technique, and we also extended our analysis to patients with paratyphoid fever, the other major cause of enteric fever in South Asia, where *S.* Paratyphi A accounts for up to 20% of cases of enteric fever [1], [2], [29]. We refer to this assay as the TPTest (typhoid and paratyphoid fever test).

## Materials and Methods

### Study population and sample collection

We enrolled individuals (n = 243) presenting for care at the Kamalapur icddr,b field station with 3–7 days of fever ≥39°C who were clinically suspected to have enteric fever for whom no alternate diagnosis was evident Kamalapur is a densely populated area in Dhaka, Bangladesh [10]. At enrollment, we collected venous blood (5 ml from young children and 10 ml from others) for microbiologic culture and antimicrobial susceptibility testing, as well as for analysis by the TPTest.

We also collected blood from the patients (n = 32) who were positive by relevant laboratory techniques for other febrile illnesses, and we analyzed these specimens using the TPTest. We also enrolled healthy controls (n = 74; 25 adults, 25 older children and 24 young children) from the same endemic zone but without any acute illness, and analyzed their blood by the TPTest [28]. All specimens were collected from the participants between August 2008 to November 2011.

### Ethics statement

This study was approved by the research review and the ethical review committees of the icddr,b and informed written consent was obtained from guardians of child participants (1–17 years) and adult participants (18–59 years) provided their own consent.

### Isolation of organisms from blood cultures

Blood (3 ml for children <5 years of age and 5 ml from others) was collected and cultured using the automated BacT/Alert method [28], followed by sub-culture on MacConkey agar, blood agar and chocolate agar plates and incubation overnight at 37°C. Organisms were identified on the basis of growth characteristics, colony morphology, microscopic examination of Gram stained smear, biochemical tests and by slide agglutination tests with *Salmonella*-specific anti-sera (Denka Sieken, Tokyo, Japan) [28], [30].

### Diagnosis of enteric fever by TPTest

Peripheral blood mononuclear cells (PBMCs) from venous blood were isolated by density gradient centrifugation on Ficoll-Isopaque (Pharmacia, Uppsala, Sweden), and obtained supernatants from cultured lymphocytes as previously described [28]. Briefly, unstimulated PBMCs were cultured at 10^7^ cells/ml in RPMI complete medium [RPMI 1640 (Gibco, Gaithersburg, MD) with 10% heat-inactivated fetal bovine serum (HyClone, Ogden, UT), 100 µg of streptomycin/ml, 100 U of penicillin/ml, 100 mM pyruvate, and 200 mM L- glutamine (Gibco) [28], [31] at 37°C in an incubator with 5% CO_2_ supply. After incubation for various times, IgA antibodies specific to *S.* Typhi membrane preparation (MP) were measured in the culture supernatants by enzyme-linked immunosorbent assay (ELISA) method as previously described [28]. Briefly, the wells of microtiter plates (Nunc F; Nunc, Denmark) were coated with MP antigen (10 µg/ml; see below for preparation). After overnight incubation at room temperature, the plates were blocked with 1% bovine serum albumin (BSA) in PBS. A 100 µl of diluted (1∶2 dilution with 0.1% BSA-PBS-Tween) culture supernatant was added to each well and incubated the plates at 37°C for 90 min. After washing with 0.05% PBS-Tween, the horseradish peroxidase conjugated antibodies to human IgA (Jackson Laboratories, Bar Harbor, ME) were added and the plates were developed with ortho-phenylene diamine (Sigma Chemical Co., St. Louis, MO) in 0.1 M sodium citrate buffer and 0.1% hydrogen peroxide and then read kinetically at 450 nm for five minutes at 19-second intervals. The maximal rate of optical density change was expressed as milli optical density absorbance units per minute (mAB/min). To avoid the discrepancy among plates, the mAB/min kinetic reaction rate of test samples was divided by that of a standard comprised of pooled convalescent phase sera previously collected from patients with documented *S.* Typhi bacteremia in Dhaka, Bangladesh, and the product was multiplied by 100 and was expressed as ELISA units [28]. The cut-off value of the TPTest was calculated as greater than the geometric mean ELISA units, plus two standard deviations, of samples from healthy endemic zone controls (a positive value was thus defined as >10 ELISA units). The initial TPTest used culture supernatants collected after 48 hours of incubation. For 20 patients, the TPTest was also carried out after incubation of lymphocytes for 24 hours as well as 48 hours. For 30 patients aged younger than 5 years, we used one ml of venous blood to perform the TPTest.

### Preparation of the *S.* Typhi antigen used in TPTest

The MP antigen used for the TPTest was prepared using the procedure described previously using *S.* Typhi strain Ty21a [28], [32]. The bacterial strain was cultivated on sheep blood agar plates and the bacteria harvested in buffer (5 mM MgCl_2_, 10 mM Tris [pH 8.0]). The bacterial suspension was sonicated for five times at 60% amplitude and centrifuged at 1400× g for 10 minutes. The supernatant was then transferred to fresh tubes and centrifuged at 14900× g for 30 minutes. The pellet was dissolved in harvest buffer and the protein content was determined by the Bio-Rad protein assay and stored at −70°C.

### Adaptation of the TPTest procedure for transfer to resource-limited settings

In an attempt to simplify the TPTest, we also assessed a number of modifications to the procedure and compared these results with the above described assay. To simplify PBMC recovery, in addition to isolating cells by density gradient centrifugation on Ficoll-Isopaque, we also isolated PBMCs using erythrocyte lysis buffer and separately using a crude leukocyte buffy coat (both approaches described below). We tested 25 specimens by all three cell separation methods for comparison. In order to assess whether incubation of PBMCs required the presence of supplemental CO_2_, we also compared TPTest results using supernatants recovered from unstimulated leukocytes cultured at 37°C in the presence and absence of 5% CO_2_. We also assessed the ability of immunodot blot assays to detect differences in samples, comparing results to the ELISA-based system.

### Separation of cells by RBC lysis

We diluted venous blood with lysing solution (0.15 M ammonium chloride; 1 mM potassium bicarbonate and .01 mM disodium EDTA) at 1∶10 dilution and mixed the sample by gently inverting the tube (BD Falcon) 3–5 times. We held the tube for 5 minutes at room temperature, and we then centrifuged the samples at 950× g for 10 minutes at 20°C. We decanted the supernatant and resuspended the cells in RPMI complete medium and counted in a haemocytometer. We re-centrifuged as above, and resuspended the isolated cells in RPMI complete medium to a concentration of 10^7^ cells/ml. We aliquoted the suspended cells into wells of tissue culture plates (F96 MicroWell Plate, Nunc) and incubated them at 37°C with and without 5% CO_2_ for 24–48 hours. We then harvested the culture suspension, centrifuged the samples at 11600× g at 20°C for 5 minutes, and collected the supernatant.

### Separation of cells by buffy coat procedure

We collected venous blood in sodium citrate tubes (Greiner bio-one, Vacuette, North America) and centrifuged these at 750× g for 5 min at 20°C. After removing the plasma from the top layer, we collected the buffy coat using a Pasteur pipette. We then washed the cells twice with phosphate-buffered saline (PBS; 10 mM; pH-7.2) and resuspended the cells in RPMI complete medium to a concentration of 10^7^ cells/ml and incubated the cells as above at 37°C with and without 5% CO_2_ for 24–48 hours. We then harvested the culture suspension, centrifuged the samples at 11600× g at 20°C for 5 minutes, and collected the supernatant.

### Assessment of TPTest ELISA system at different time points of illness

To assess whether the TPTest remained positive over the course of the illness and recovery, we also assessed TPTest reactivity on days 7 and 21 in 38 patients who were positive for the TPTest (>10 ELISA unit) at the day of enrollment. Among these 38 patients, 18 patients were positive by both blood culture (17 patients had *S.* Typhi bacteremia and one had *S.* Paratyphi A bacteremia) and TPTest, and rest of the patients were positive only by the TPTest.

To assess the reproducibility of the TPTest, we also randomly selected 10 adult patients from the 243 enrolled who were negative for both blood culture and TPTest on enrollment, and repeated the TPTest on days 7 and day 21 after enrollment.

### Immunodot blot assay

To preliminarily evaluate whether an immunodot blot system could be developed for use in settings lacking ELISA capability, we also assessed an immunodot blot-based approach. To do this, we divided strips of 30×30 cm Osmonics NitroBind 0.45 µm Transfer Membrane (Krackeler Scientific, Inc, Albany, New York) into 0.35 by 0.35 cm squares. We soaked these strips in PBS and allowed the membranes to dry before coating them with 1 µl of MP antigen (1 mg/ml MP antigen dissolved in PBS), 1 µl of AffiniPure Goat Anti-Human IgG, F(ab′)_2_ Fragment (1.8 mg/ml dissolved in deionized water) as a positive control or 1 µl of lipoolysaccharide (LPS) of *Vibrio cholerae* O1 X-25049 strain (Ogawa) (1 mg/ml dissolved in PBS) as a negative control at room temperature for 5 minutes. We then blocked the membranes with 1% bovine serum albumin in PBS at room temperature for 20 minutes using slow shaking (230 rpm; Gyrotory Water Bath Shaker; New Brunswick Scientific). We discarded the blocking solution and washed the membranes twice with PBS. To assess immuno-reactivity, we added lymphocyte supernatants diluted 1∶2 with 0.1% BSA-PBS-0.05% Tween to membranes and incubated these for 3 hours at room temperature with slow shaking (230 rpm; Gyrotory Water Bath Shaker; New Brunswick Scientific). We then washed the membranes five times with PBS-Tween (0.05%) and once with PBS. We then incubated the membranes with rabbit anti human IgA conjugated to horse radish peroxidase (Jackson Immnoreaserch Laboratories, Inc. West Grove. USA at a 1∶500 dilution in 0.1% BSA-PBS-Tween) for 1.5 hours at room temperature with shaking. We washed the membrane five times with PBS-Tween (0.05%) and once with PBS. We developed the membranes by adding H_2_O_2_- 4-chloro- 1-naphthol, prepared by dissolving 1.7 ml 4-chloro- 1-napthol (3 mg/ml in 99.9% methanol) in 8.3 ml of Tris buffered saline (TBS; 20 mM Tris; 0.5 M NaCl; pH-7.5), to which 0.015% H_2_O_2_ was added immediately before use. Reactivity was read at 5 minutes. Membranes were then washed with water and air dried. We considered immunodots positive if reactivity could be seen with the naked eye after membranes had dried. Dots were independently read by two technicians with 100% concordance.

### Statistical analysis

We used SigmaStat (version3.1) and Prism4, EpiInfo (version 2000; Centers for Disease Control and Prevention) for data management, analysis, and graphical presentation. We performed statistical evaluation of differences among groups by using the Mann-Whitney U test and considered results statistically significant if p<0.05. We have calculated the sensitivity and specificity with 95% confidence interval of the TPTest using two by two tables.

## Results

### Blood culture results

Out of 243 enrolled patients suspected of having enteric fever, 59 were adults (18–59 years of age), 108 were older children (6–17 years of age) and 76 patients were young children (1–5 years of age). We isolated *S.* Typhi or *S.* Paratyphi A in the blood of 27 (11%) and 12 (5%) patients, respectively. Among 27 patients bacteremic with *S.* Typhi, 18 (67%) were older children, 7 (26%) were younger children, and 2 (7%) were adults. Among 12 patients bacteremic with *S.* Paratyphi A, 9 (75%) were older children and the remaining 3 (25%) were young children. The initiation of antimicrobial therapy was at the discretion of the attending physician; however, many individuals with suspected enteric fever received empiric treatment with a third generation cephalosporin such as oral cefixime or parenteral ceftriaxone for 14–21 days, including all patients subsequently reactive in the TPTest (see below). All patients recovered, and there were no deaths or severe complications in this cohort.

### Categories of the study participants

For analyses, we categorized the individuals studied into four groups (Table 1) as follows- Group I- patients with suspected enteric fever and confirmed bacteremia with *S.* Typhi or *S.* Paratyphi A organisms (n = 39); Group II- patients with clinical characteristics compatible with enteric fever, but in whom blood cultures remained negative (n = 204); Group III- patients confirmed by relevant methods to have other illnesses (n = 32; dengue (n = 15), leptospirosis (n = 1), *Streptococcus pneumoniae* bacteremia (n = 1), tuberculosis (n = 7), visceral leishmaniasis (n = 5), respiratory tract infection (n = 1), acute hepatitis B infection (n = 1) and acute hepatitis E virus (n = 1) infection. Blood culture was carried out in patients with other illnesses except those with tuberculosis and visceral leishmaniasis. Group IV- healthy endemic zone controls (n = 74).

**Table 1:** TPTest results in different study groups.

Group	Characteristics	No. of individuals	TPTest positive*	TPTest negative
Group I	Clinically suspected enteric fever, blood culture grew *S.* Typhi	27	27	0
	Clinically suspected enteric fever, blood culture grew *S.* Paratyphi A	12	12	0
Group II	Clinically suspected enteric fever but blood culture negative	204	44	160
Group III	Confirmed illness other than enteric fever	32	3	29
Group IV	Healthy control	74	0	74

* >10 ELISA unit was considered a positive TPTest (see text).

### Results of the TPTest

As shown in table 1, all patients with confirmed *S.* Typhi or *S.* Paratyphi A bacteremia (Group I) were positive in the TPTest. Among 204 suspected of having enteric fever but blood culture negative (Group II), 44 patients were positive by the TPTest. The TPTest was positive in 3 out of 32 patients with other illnesses (Group III). Of these three TPTest positive patients, one was bacteremic for *S. pneumonie*, one had acute dengue fever, and one had visceral leishmaniasis. None of the 74 healthy endemic zone controls (Group IV) were positive by the TPTest. One ml of blood was sufficient for carrying out the TPTest in 30 patients less than five years of age.

### Clinical characteristics of patients with suspected enteric fever who were blood culture positive compared with those who were blood culture negative but TPTest positive

Clinical characteristics of patients in these groups are presented in Table 2. Of patients with confirmed *S.* Typhi or *S.* Paratyphi A bacteremia, 21 were male and 18 female. Of patients with a positive TPTest and negative blood culture, 22 were male and 22 female. Of the 243 patients with suspected enteric fever, 23 (9.5%) individuals had taken antibiotics before enrollment. Of these, three patients were positive by blood culture, whereas eight individuals were positive only by the TPTest. The number of young children (<5 years of age) between the blood culture and TPTest positive group (39) and the blood culture negative but TPTest positive group (44) was significantly different (p = 0.01), with negative blood cultures being more common in young children. No other differences were detected among the groups.

**Table 2:** Clinical characteristics of study participants.

Characteristics	Patients with confirmed *S.* Typhi or *S.* Paratyphi A bacteremia (Total no. = 39) no. (%)	Clinically suspected enteric fever, blood culture negative but TPTest positive (Total no. = 44) no. (%)
Number of male	21 (54)	22 (50)
Children <5 years of age	10 (26)	23 (52)^a^
Duration of fever, days (median) (25th, 75th percentile)	4 (3, 5)	4 (3, 5)
Temperature in °C (median) (25th, 75th percentile)	39.2 (39.1, 39.5)	39.2 (39.0, 39.4)
Prior antibiotic intake	3 (8)	8 (18)
Hospitalization	2 (5)	0 (0)
Headache	6 (15)	12 (27)
Coated tongue	10 (26)	11 (25)
Myalgias	6 (15)	11 (25)
Abdominal pain	7 (18)	13 (30)
Tender abdomen	3 (8)	0 (0)
Constipation	4 (10)	3 (7)
Diarrhea	3 (8)	7 (16)
Loss of appetite	17 (44)	14 (32)

a Significantly different (p<0.05) when compared blood culture confirmed bacteremia.

### Sensitivity and specificity of the TPTest

The sensitivity and specificity of the TPTest are shown in Table 3. Considering blood culture-confirmed cases as positive and the patients with other illnesses and healthy controls as negative for enteric fever , the sensitivity and specificity of the TPTest were 39/39 (100%) and 103/106 (97%; 95% confidence interval, CI: 94–100), respectively. Considering blood culture-confirmed cases as positive and blood culture-negative patients as enteric fever negative, the TPTest was 39/39 (100%) sensitive and 160/204 (78%, 95% CI: 73–84) specific. Considering blood culture-confirmed cases as positive and the patients negative by blood culture, patients with other illness and healthy controls as enteric fever negative, the sensitivity and specificity of the TPTest were 39/39 (100%) and 263/310 (85%, 95% CI: 81–89).

**Table 3:** Sensitivity, specificity (with 95% confidence intervals in parenthesis) of TPTest for diagnosing enteric fever.

Parameters	Sensitivity^a^ (%)	Specificity^a^ (%)
Considering blood culture-confirmed cases as positive and the patients with other illness and healthy controls as negative for enteric fever	39/39 (100)	103/106 (97, CI: 94–100 )
Considering blood culture-confirmed cases as positive and blood culture-negative patients as enteric fever negative	39/39 (100)	160/204 (78, CI: 73–84)
Considering blood culture-confirmed cases as positive and the patients negative by blood culture, patients with other illness and healthy controls as enteric fever negative	39/39 (100)	263/310 (85, CI: 81–89)

a Calculated using a two by two table.

### Comparison of the results of the TPTest using lymphocyte supernatants harvested after 24 and 48 hours of culture

**Figure 1:**
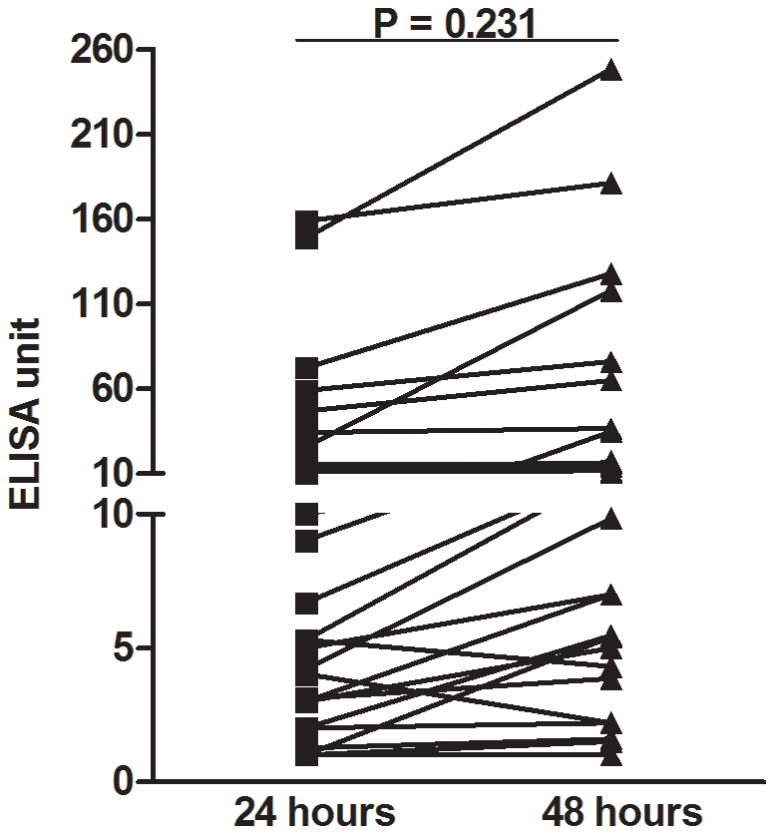
Effect on TPTest results of different incubation periods of cells. The TPTest was done using lymphocyte supernatants after 24 and 48 hours incubation of peripheral blood mononuclear cells (PBMCs) at 37°C with supplemental CO_2_. >10 ELISA units was considered a positive TPTest.

For 28 patients, we performed the TPTest using lymphocyte culture supernatants collected at both 24 and 48 hours (p = 0.231) of incubation. Among these, 14 patients were positive for the TPTest (>10 ELISA unit) following 48 hours of incubation. Of these, 9 (64%) test results were positive following 24 hours (and remained positive after 48 hours), and 5 (36%) were negative at 24 hours but became positive at 48 hours of incubation (Figure 1). All negative samples using fluid collected at 48 hours (n = 14) were also negative using the 24 hour supernatants.

### Results of the simplified TPTest

**Figure 2:**
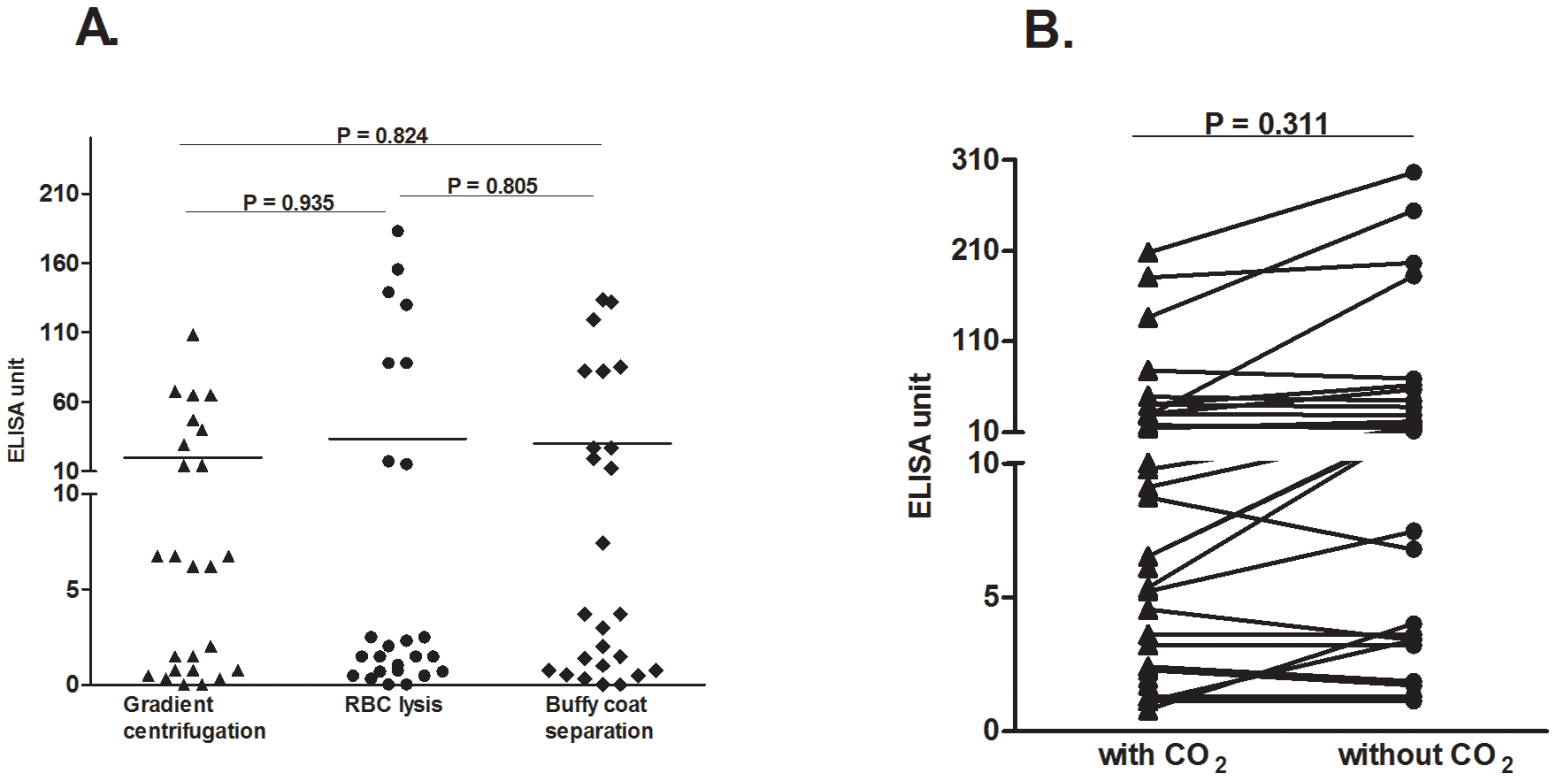
Comparison of TPTest results using various techniques. (**A**) The TPTest was carried out using leukocytes recovered by various techniques: density gradient centrifugation, erythrocyte lysis buffer, and buffy coat separation. The Mann-Whitney U test was used to compare the responses of different blood cell separation methods. RBC, red blood cell. (**B**) Comparison of TPTest results using peripheral blood mononuclear cells incubated at 37°C in the presence or absence of supplemental CO_2_. >10 ELISA units was considered a positive TPTest (see text).

**Figure 3:**
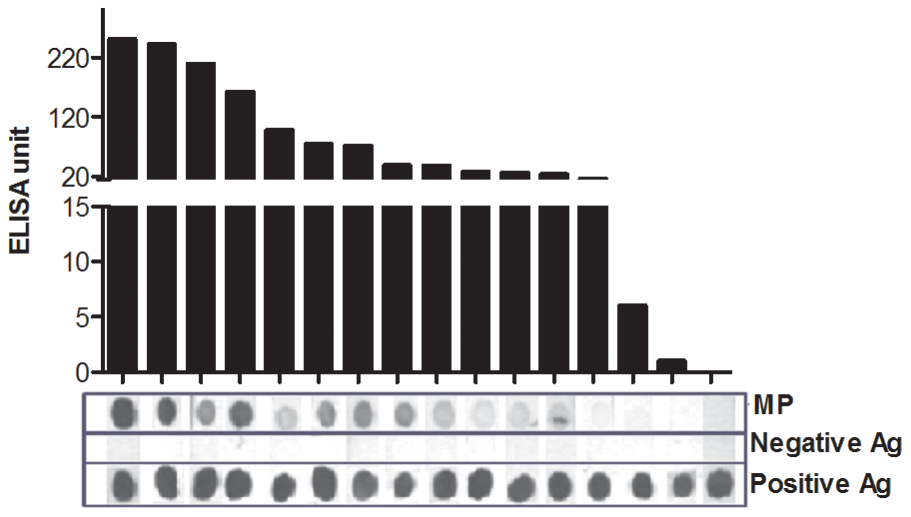
Comparison of *Salmonella* specific IgA responses using ELISA on lymphocyte secretions versus immunodot blot approaches. Anti-*Salmonella* membrane preparation (MP) specific IgA responses using ELISA on lymphocyte secretions was compared to immunodot blot approaches. ELISA positive defined as >10 ELISA units (see text). Immunodot blot approaches positive in settings of ≥16 ELISA units. Positive antigen (Ag): AffiniPure Goat Anti-Human IgG, F(ab′)_2_ Fragment. Negative antigen (Ag): LPS of *Vibrio cholerae* O1 X-25049 strain (Ogawa).

We found no significant differences (p = 0.805 to p = 0.935) comparing TPTest results using PBMCs separated by RBC lysis or buffy coat procedure, compared to cells separated by density gradient centrifugation (Figure 2A). Similarly, we did not detect significant differences (p = 0.311) in MP-IgA values in lymphocyte supernatant fluid when the PBMCs were cultured in an incubator with supplemental 5% CO_2_ or without CO_2_ (Figure 2B). Using the immunodot blot assay, we detected MP-IgA antibodies in lymphocyte supernatants utilizing nitrocellulose membranes (Figure 3). We tested the immunodot blot assay in 23 positive specimens (>10 ELISA unit in TPTest) and in 5 negative specimens (<10 ELISA unit in TPTest). TPTest specimens with values ≥16 ELISA units were detected by the immunodot blot assay. None of the specimens negative by the TPTest specimens was positive by the dot blot method.

### Duration of TPTest responses

To assess the duration of TPTest responses, we collected blood from later stages of illness (days 7 and 21) from 38 patients with a positive TPTest at the time of presentation. Of these, 32 (84%) patients converted to a negative result by day 21∶18 (56%) patients became negative by day 7, and the additional 14 (44%) became negative by day 21, suggested that the TPTest assesses a transient response in enteric fever. Among six patients who failed to revert to negative by day 21, five (83%) were positive for *S.* Typhi bacteremia and one was positive by the TPTest only.

Among 10 patients negative by TPTest at clinical presentation, all 10 remained negative at days 7 and 21.

## Discussion

There is an urgent need for an improved diagnostic assay for enteric fever [25]. Microbiologic culturing of peripheral blood requires 3–10 ml and is only 30–70% sensitive [1], [3], [10], [33]. Obtaining this volume of blood from young children for a single test is often problematic. The low sensitivity in part reflects the low bacterial burden in peripheral blood during enteric fever (median 0.1–1.0 CFU/ml) [34], the less than optimal volume of blood usually obtained in clinical settings, especially from young children, and the inhibitory effects of insufficient antibiotics that many patients ingest before seeking medical care. Microbiologic analysis of a bone marrow aspirate is often considered a “gold standard”, but aspiration of marrow is not clinically practical or acceptable in many settings, and microbiologic culturing of marrow in patients who ingest antibiotics prior to clinical presentation may also be falsely negative. Since the increased sensitivity of marrow is due to the approximately ∼10-fold higher bacterial load in marrow versus peripheral blood [17], [22], [35], culturing a larger volume of blood should be equivalent to culturing marrow, but fundamentally the problem inherent in all diagnostic assays for enteric fever based on microbiologic detection of bacteria is one of a very low clinically accessible bacterial burden.

To overcome such shortcomings, assays based on amplification of bacterial nucleic acids have been the focus of many investigations [25], [36]–[38], since theoretically, such an approach could have the ability to detect a single organism (living or dead). Unfortunately, the development of such an approach has also not yet been successful. The reasons for this are not clear, but may also relate to the requirement once again for relatively large volumes of blood, the difficulty of detecting extremely low levels of bacterial DNA and/or RNA in the blood, especially in the presence of comparably overwhelming amounts of eukaryotic DNA and RNA, and the effects of reaction inhibitors present in human blood [39]–[41].

Since assays based on directly detecting *S.* Typhi and *S.* Paratyphi have such significant shortcomings, alternative approaches have focused on detecting human responses to the bacteria, and indeed, such approaches are the bases of current and the commercially available serum antibody-based Widal and anti-lipopolysaccharide and anti-*Salmonella enterica* assays [14], [42], [43]. Unfortunately, the utility of these assays has been markedly limited in areas of the world endemic for enteric fever, since many individuals in these zones have been previously exposed to *S.* Typhi and *S.* Paratyphi, and have persistently positive assays from previous exposure [44]–[46]. Additional approaches currently being evaluated include assessing cytokine profiles in the blood of patients in the acute phase of enteric fever, or cellular responses during the acute stage of infection, including assessing interferon-gamma release assays [47].

Because of the shortcomings of current enteric fever diagnostic assays, we were particularly interested in assessing whether we could pursue an alternative approach and take advantage of the fact that following recent infection activated antigen-specific lymphocytes transiently circulate in the peripheral circulation and could be detected there [28]. In support of this, we have previously found that measuring anti-*Salmonella* serum IgA antibodies in patients was only sensitive for detecting *S.* Typhi bacteremia in 81%, but that we could improve those results by harvesting PBMCs and analyzing antibodies following *ex vivo* culturing of the recovered lymphocytes [28]. Importantly, we found that such an approach did not require *in vitro* stimulation of the recovered lymphocytes, presumably because the cells had already been stimulated in vivo during the acute infection. We hypothesized that detecting such antigen-specific circulating lymphocytes would improve specificity, since stimulated lymphocytes should *de facto* be most prominent in individuals actively infected with *S.* Typhi and *S.* Paratyphi. We further hypothesized that such an approach might be particularly useful in enteric fever endemic zones, where many individuals might be immunologically primed to mount a detectable response without *ex vivo* antigen-specific stimulation. Indeed, using such an approach, we previously found in a small pilot study in Dhaka, Bangladesh, a typhoid fever endemic area, that we could identify 100% of patients who were bacteremic with *S.* Typhi, by examining supernatants recovered from harvested and unstimulated lymphocytes for IgA antibodies reactive with an *S.* Typhi membrane preparation [28].

In this current and larger study, we extend these findings and confirm that such an approach not only identifies all patients bacteremic with *S.* Typhi, but also all patients bacteremic with *S.* Paratyphi A using the same membrane preparation (MP) antigen recovered from *S.* Typhi Ty21a strain and using similar specimens. Previously performed mass spectrometric analysis reveals that MP contains many *Salmonella eneterica* proteins related to energy metabolism, virulence and pathogenesis [48]. The fact that the majority of patients with a positive TPTest became negative by day 21 also supports the assumption that the assay detects recently stimulated and transiently migrating lymphocytes.

Recognizing that enteric fever is largely a disease of resource-limited settings, we also tried a number of simplifications to our assay approach. Our data suggests that cells can be recovered from peripheral blood using an erythrocyte lysis buffer, buffy coat preparation and incubated at 37°C with no supplemental CO_2_, with similar results. We also found that supernatants could be assayed at both 24 and 48 hours, to identify positive responses as quickly as possible. We did not find any significant difference in severity of disease or blood culture positivity among patients who were TPTest positive and those who were not positive at the early time point but then became positive at the later time point. We could accurately identify the vast majority of positive TPTest responses using an immunodot-based assay as well as the ELISA format, suggesting that development of a lateral flow device or dipstick-based assay based on looking for antigen-specific IgA in lymphocyte supernatants could be possible.

Our study has a number of limitations. The most important is that there is no true practical gold standard for enteric fever against which we can assess the TPTest. Serologic tests are not helpful in enteric fever endemic zones, nucleotide amplification assays are currently not sensitive, reproducible, standardized or useful, microbiologic culturing of blood is not sensitive, and microbiologic culturing of bone marrow is not clinically acceptable. In this study, if we defined our true positives as individuals with confirmed bacteremia, and defined our true negatives as those with confirmed “other diagnoses” including healthy control, the TPTest would have a sensitivity of 100% and specificity of 97% (95% CI: 94–100). At the other end of the spectrum, if we defined our true positives as individuals with suspected enteric fever and confirmed bacteremia, and defined our true negatives as individuals with suspected enteric fever but a negative blood culture alone or a negative culture and patients with other confirmed illnesses and healthy controls, the TPTest would retain a sensitivity of 100%, but specificity would fall to 78% (95% CI: 73–84) and 85% (95% CI: 81–89), respectively, still a significant advance over current assays.

False positive results were seen in three patients with other illnesses. Among these, one patient was categorized as an acute dengue infection based on a positive IgM for acute dengue fever ; however, this individual also had a positive Widal test with a TH antibody titer ≥1∶320. This patient was treated with cefixime by the clinical service and recovered. The other two false positive patients had confirmed *S. pneumonie* bacteremia or a positive antibody consistent with a diagnosis of visceral leishmaniasis; these patients were treated accordingly and recovered. The TPTest appears to have been falsely positive in these two acutely febrile individuals, although the chronic *S.* typhi or *S.* Paratyphi carrier status of these two individuals is unknown; an issue that will require further evaluation.

Recognizing that blood culture sensitivity for enteric fever often approximates 50% in clinical practice, our identification of roughly equal numbers of patients with a positive blood culture and positive TPTest (n = 39) and positive TPTest and negative blood culture (n = 44), suggests that some or all of these patients may actually have had enteric fever, especially since these two groups were largely clinically indistinguishable and that both clinically improved following therapy that targeted enteric fever. Of interest is the observation that a positive TPTest but negative blood culture was more common in children under 5 years of age compared to older individuals. Although young children have a higher bacterial load of *Salmonella enterica* than older individuals in peripheral blood [34], in our experience the smallest volume of blood is often collected for microbiologic analysis in young children. Of note, the TPTest is performed on 1 ml of blood, an attribute that makes the assay attractive for use in young children.

At present, the TPTest has significant shortcomings. It is not point-of-care, although our data suggest that it could be performed by a rudimentary laboratory with minimally trained personnel. The TPTest also does not provide microbiologic identification to the species level (*S.* Typhi versus *S.* Paratyphi) and does not provide antimicrobial susceptibility profiles. Similarly, the assay currently uses a crude membrane preparation of *S.* Typhi as target antigen, and pooled convalescent blood to standardize the ELISA, both of which are areas of ongoing investigation. Also, we have not yet tested this method in S. Typhi and S. Paratyphi chronic carriers. Despite these shortcomings, our results suggest that an assay based on detecting anti-*Salmonella* IgA antibodies produced by transiently circulating and activated lymphocytes may hold significant promise as a platform approach to identify individuals with enteric fever, including in endemic zones. Our data also suggest that such an approach could be performed in resource-limited settings, requiring only 1 ml of blood, facilitating its use in young children, and with results available within 24–48 hours. Of note, the TPTest could be used either as a clinical diagnostic tool to inform treatment approaches for individual patients, as well as a surveillance tool to estimate disease burden and assist in control programs targeting enteric fever. As such, we believe these results support further development of this diagnostic approach.

## Supporting Information

Figure S1 **STARD flowchart of the study.**
(TIF)

Table S1 **STARD checklist of the study.**
(DOC)

## References

[1] Crump JA, Luby SP, Mintz ED (2004) The global burden of typhoid fever. Bull World Health Organ 82: 346–353. 15298225 PMC2622843

[2] Fangtham M, Wilde H (2008) Emergence of *Salmonella* paratyphi A as a major cause of enteric fever: need for early detection, preventive measures, and effective vaccines. J Travel Med 15: 344–350. 19006508 10.1111/j.1708-8305.2008.00237.x

[3] Butler T, Islam A, Kabir I, Jones PK (1991) Patterns of morbidity and mortality in typhoid fever dependent on age and gender: review of 552 hospitalized patients with diarrhea. Rev Infect Dis 13: 85–90. 2017639 10.1093/clinids/13.1.85

[4] Sinha A, Sazawal S, Kumar R, Sood S, Reddaiah VP, et al (1999) Typhoid fever in children aged less than 5 years. Lancet 354: 734–737. 10475185 10.1016/S0140-6736(98)09001-1

[5] Nakachi S, Nakamura T, Agha N, Daud S, Mohd S, et al (2003) Clinical features and early diagnosis of typhoid fever emphasizing usefulness of detecting mesenteric lymphadenopathy with ultrasound as diagnostic method. Southeast Asian J Trop Med Public Health 34 Suppl 2: 153–157. 19230587

[6] Maskey AP, Day JN, Phung QT, Thwaites GE, Campbell JI, et al (2006) *Salmonella enterica* serovar Paratyphi A and S. *enterica* serovar Typhi cause indistinguishable clinical syndromes in Kathmandu, Nepal. Clin Infect Dis 42: 1247–1253. 16586383 10.1086/503033

[7] Crump JA, Mintz ED (2010) Global trends in typhoid and paratyphoid Fever. Clin Infect Dis 50: 241–246. 20014951 10.1086/649541 PMC2798017

[8] Ochiai RL, Acosta CJ, Danovaro-Holliday MC, Baiqing D, Bhattacharya SK, et al (2008) A study of typhoid fever in five Asian countries: disease burden and implications for controls. Bull World Health Organ 86: 260–268. 18438514 10.2471/BLT.06.039818 PMC2647431

[9] Saha SK, Baqui AH, Hanif M, Darmstadt GL, Ruhulamin M, et al (2001) Typhoid fever in Bangladesh: implications for vaccination policy. Pediatr Infect Dis J 20: 521–524. 11368111 10.1097/00006454-200105000-00010

[10] Brooks WA, Hossain A, Goswami D, Nahar K, Alam K, et al (2005) Bacteremic typhoid fever in children in an urban slum, Bangladesh. Emerg Infect Dis 11: 326–329. 15752457 10.3201/eid1102.040422 PMC3320465

[11] Hoffman SL, Punjabi NH, Kumala S, Moechtar MA, Pulungsih SP, et al (1984) Reduction of mortality in chloramphenicol-treated severe typhoid fever by high-dose dexamethasone. N Engl J Med 310: 82–88. 6361558 10.1056/NEJM198401123100203

[12] Bitar R, Tarpley J (1985) Intestinal perforation in typhoid fever: a historical and state-of-the-art review. Rev Infect Dis 7: 257–271. 3890098 10.1093/clinids/7.2.257

[13] Parry CM, Hien TT, Dougan G, White NJ, Farrar JJ (2002) Typhoid fever. N Engl J Med 347: 1770–1782. 12456854 10.1056/NEJMra020201

[14] Chew SK, Cruz MS, Lim YS, Monteiro EH (1992) Diagnostic value of the Widal test for typhoid fever in Singapore. J Trop Med Hyg 95: 288–291. 1495127

[15] Parry CM, Hoa NT, Diep TS, Wain J, Chinh NT, et al (1999) Value of a single-tube widal test in diagnosis of typhoid fever in Vietnam. J Clin Microbiol 37: 2882–2886. 10449469 10.1128/jcm.37.9.2882-2886.1999 PMC85403

[16] Olsen SJ, Pruckler J, Bibb W, Nguyen TM, Tran MT, et al (2004) Evaluation of rapid diagnostic tests for typhoid fever. J Clin Microbiol 42: 1885–1889. 15131144 10.1128/JCM.42.5.1885-1889.2004 PMC404619

[17] Gilman RH, Terminel M, Levine MM, Hernandez-Mendoza P, Hornick RB (1975) Relative efficacy of blood, urine, rectal swab, bone-marrow, and rose-spot cultures for recovery of *Salmonella* typhi in typhoid fever. Lancet 1: 1211–1213. 48834 10.1016/s0140-6736(75)92194-7

[18] Farooqui BJ, Khurshid M, Ashfaq MK, Khan MA (1991) Comparative yield of *Salmonella* typhi from blood and bone marrow cultures in patients with fever of unknown origin. J Clin Pathol 44: 258–259. 2013632 10.1136/jcp.44.3.258 PMC496954

[19] Choo KE, Oppenheimer SJ, Ismail AB, Ong KH (1994) Rapid serodiagnosis of typhoid fever by dot enzyme immunoassay in an endemic area. Clin Infect Dis 19: 172–176. 7948526 10.1093/clinids/19.1.172

[20] Gupta AK, Rao KM (1979) Simultaneous detection of *Salmonella* typhi antigen and antibody in serum by counter-immunoelectrophoresis for an early and rapid diagnosis of typhoid fever. J Immunol Methods 30: 349–353. 512365 10.1016/0022-1759(79)90017-6

[21] Bhutta ZA, Mansurali N (1999) Rapid serologic diagnosis of pediatric typhoid fever in an endemic area: a prospective comparative evaluation of two dot-enzyme immunoassays and the Widal test. Am J Trop Med Hyg 61: 654–657. 10548305 10.4269/ajtmh.1999.61.654

[22] Gasem MH, Smits HL, Goris MG, Dolmans WM (2002) Evaluation of a simple and rapid dipstick assay for the diagnosis of typhoid fever in Indonesia. J Med Microbiol 51: 173–177. 11865843 10.1099/0022-1317-51-2-173

[23] Naheed A, Ram PK, Brooks WA, Mintz ED, Hossain MA, et al (2008) Clinical value of Tubex and Typhidot rapid diagnostic tests for typhoid fever in an urban community clinic in Bangladesh. Diagn Microbiol Infect Dis 61: 381–386. 18501549 10.1016/j.diagmicrobio.2008.03.018

[24] Siba V, Horwood PF, Vanuga K, Wapling J, Sehuko R, et al (2012) The Evaluation of serological diagnostic tests for Typhoid Fever in Papua New Guinea using a composite reference standard. Clin Vaccine Immunol 19: 1833–7. 22993409 10.1128/CVI.00380-12 PMC3491554

[25] Parry CM, Wijedoru L, Arjyal A, Baker S (2011) The utility of diagnostic tests for enteric fever in endemic locations. Expert Rev Anti Infect Ther 9: 711–725. 21692675 10.1586/eri.11.47

[26] Olopoenia LA, King AL (2000) Widal agglutination test - 100 years later: still plagued by controversy. Postgrad Med J 76: 80–84. 10644383 10.1136/pmj.76.892.80 PMC1741491

[27] Espersen F, Hoiby N, Hertz JB (1980) Cross-reactions between *Salmonella* typhi and 24 other bacterial species. Acta Pathol Microbiol Scand B 88: 243–248. 7415844 10.1111/j.1699-0463.1980.tb02635.x

[28] Sheikh A, Bhuiyan MS, Khanam F, Chowdhury F, Saha A, et al (2009) *Salmonella enterica* serovar Typhi-specific immunoglobulin A antibody responses in plasma and antibody in lymphocyte supernatant specimens in Bangladeshi patients with suspected typhoid fever. Clin Vaccine Immunol 16: 1587–1594. 19741090 10.1128/CVI.00311-09 PMC2772369

[29] Sheikh A, Charles RC, Rollins SM, Harris JB, Bhuiyan MS, et al (2010) Analysis of *Salmonella enterica* serotype paratyphi A gene expression in the blood of bacteremic patients in Bangladesh. PLoS Negl Trop Dis 4: e908. 21151879 10.1371/journal.pntd.0000908 PMC2998432

[30] Talawadekar NN, Vadher PJ, Antani DU, Kale VV, Kamat SA (1989) Chloramphenicol resistant *Salmonella* species isolated between 1978 and 1987. J Postgrad Med 35: 79–82. 2621666

[31] Qadri F, Ryan ET, Faruque AS, Ahmed F, Khan AI, et al (2003) Antigen-specific immunoglobulin A antibodies secreted from circulating B cells are an effective marker for recent local immune responses in patients with cholera: comparison to antibody-secreting cell responses and other immunological markers. Infect Immun 71: 4808–4814. 12874365 10.1128/IAI.71.8.4808-4814.2003 PMC165990

[32] Bhuiyan TR, Qadri F, Saha A, Svennerholm AM (2009) Infection by Helicobacter pylori in Bangladeshi children from birth to two years: relation to blood group, nutritional status, and seasonality. Pediatr Infect Dis J 28: 79–85. 19116602 10.1097/INF.0b013e31818a5d9d

[33] Wain J, Pham VB, Ha V, Nguyen NM, To SD, et al (2001) Quantitation of bacteria in bone marrow from patients with typhoid fever: relationship between counts and clinical features. J Clin Microbiol 39: 1571–1576. 11283089 10.1128/JCM.39.4.1571-1576.2001 PMC87972

[34] Wain J, Diep TS, Ho VA, Walsh AM, Nguyen TT, et al (1998) Quantitation of bacteria in blood of typhoid fever patients and relationship between counts and clinical features, transmissibility, and antibiotic resistance. J Clin Microbiol 36: 1683–1687. 9620400 10.1128/jcm.36.6.1683-1687.1998 PMC104900

[35] Akoh JA (1991) Relative sensitivity of blood and bone marrow cultures in typhoid fever. Trop Doct 21: 174–176. 1746041 10.1177/004947559102100415

[36] Song JH, Cho H, Park MY, Na DS, Moon HB, et al (1993) Detection of *Salmonella* typhi in the blood of patients with typhoid fever by polymerase chain reaction. J Clin Microbiol 31: 1439–1443. 8314983 10.1128/jcm.31.6.1439-1443.1993 PMC265558

[37] Klouche M, Schroder U (2008) Rapid methods for diagnosis of bloodstream infections. Clin Chem Lab Med 46: 888–908. 18624614 10.1515/CCLM.2008.157

[38] Dick MH, Guillerm M, Moussy F, Chaignat CL (2012) Review of two decades of cholera diagnostics - how far have we really come? PLoS Negl Trop Dis 6: e1845. 23071851 10.1371/journal.pntd.0001845 PMC3469466

[39] Frankel G (1994) Detection of *Salmonella* typhi by PCR. J Clin Microbiol 32: 1415. 10.1128/jcm.32.5.1415-.1994 PMC263720 8051283

[40] Kumar G, Pratap CB, Mishra OP, Kumar K, Nath G (2012) Use of urine with nested PCR targeting the flagellin gene (fliC) for diagnosis of typhoid fever. J Clin Microbiol 50: 1964–1967. 22493333 10.1128/JCM.00031-12 PMC3372149

[41] Hatta M, Smits HL (2007) Detection of *Salmonella* typhi by nested polymerase chain reaction in blood, urine, and stool samples. Am J Trop Med Hyg 76: 139–143. 17255243

[42] Herath HM (2003) Early diagnosis of typhoid fever by the detection of salivary IgA. J Clin Pathol 56: 694–698. 12944555 10.1136/jcp.56.9.694 PMC1770051

[43] Cherian T, Sridharan G, Mohandas V, John TJ (1990) Prevalence of *Salmonella* typhi O and H antibodies in the serum of infants and preschool children. Indian Pediatr 27: 293–294. 2351452

[44] Kalhan R, Kaur I, Singh RP, Gupta HC (1998) Rapid diagnosis of typhoid fever. Indian J Pediatr 65: 561–564. 10773905 10.1007/BF02730895

[45] Saha SK, Ruhulamin M, Hanif M, Islam M, Khan WA (1996) Interpretation of the Widal test in the diagnosis of typhoid fever in Bangladeshi children. Ann Trop Paediatr 16: 75–78. 8787370 10.1080/02724936.1996.11747807

[46] Willke A, Ergonul O, Bayar B (2002) Widal test in diagnosis of typhoid fever in Turkey. Clin Diagn Lab Immunol 9: 938–941. 12093703 10.1128/CDLI.9.4.938-941.2002 PMC120044

[47] Sheikh A, Khanam F, Sayeed MA, Rahman T, Pacek M, et al (2011) Interferon-gamma and proliferation responses to *Salmonella enterica* Serotype Typhi proteins in patients with S. Typhi Bacteremia in Dhaka, Bangladesh. PLoS Negl Trop Dis 5: e1193. 21666798 10.1371/journal.pntd.0001193 PMC3110156

[48] Sheikh A, Charles RC, Sharmeen N, Rollins SM, Harris JB, et al (2011) In vivo expression of *Salmonella enterica* serotype Typhi genes in the blood of patients with typhoid fever in Bangladesh. PLoS Negl Trop Dis 5: e1419. 22180799 10.1371/journal.pntd.0001419 PMC3236720

