# Supplementary figures and images for: Evaluation of a Typhoid/Paratyphoid Diagnostic Assay (TPTest) Detecting Anti-Salmonella IgA in Secretions of Peripheral Blood Lymphocytes in Patients in Dhaka, Bangladesh
Source: PLoS Negl Trop Dis. 2013 Jul 11;7(7):e2316. doi: 10.1371/journal.pntd.0002316 (PMC3708850; doi:10.1371/journal.pntd.0002316)

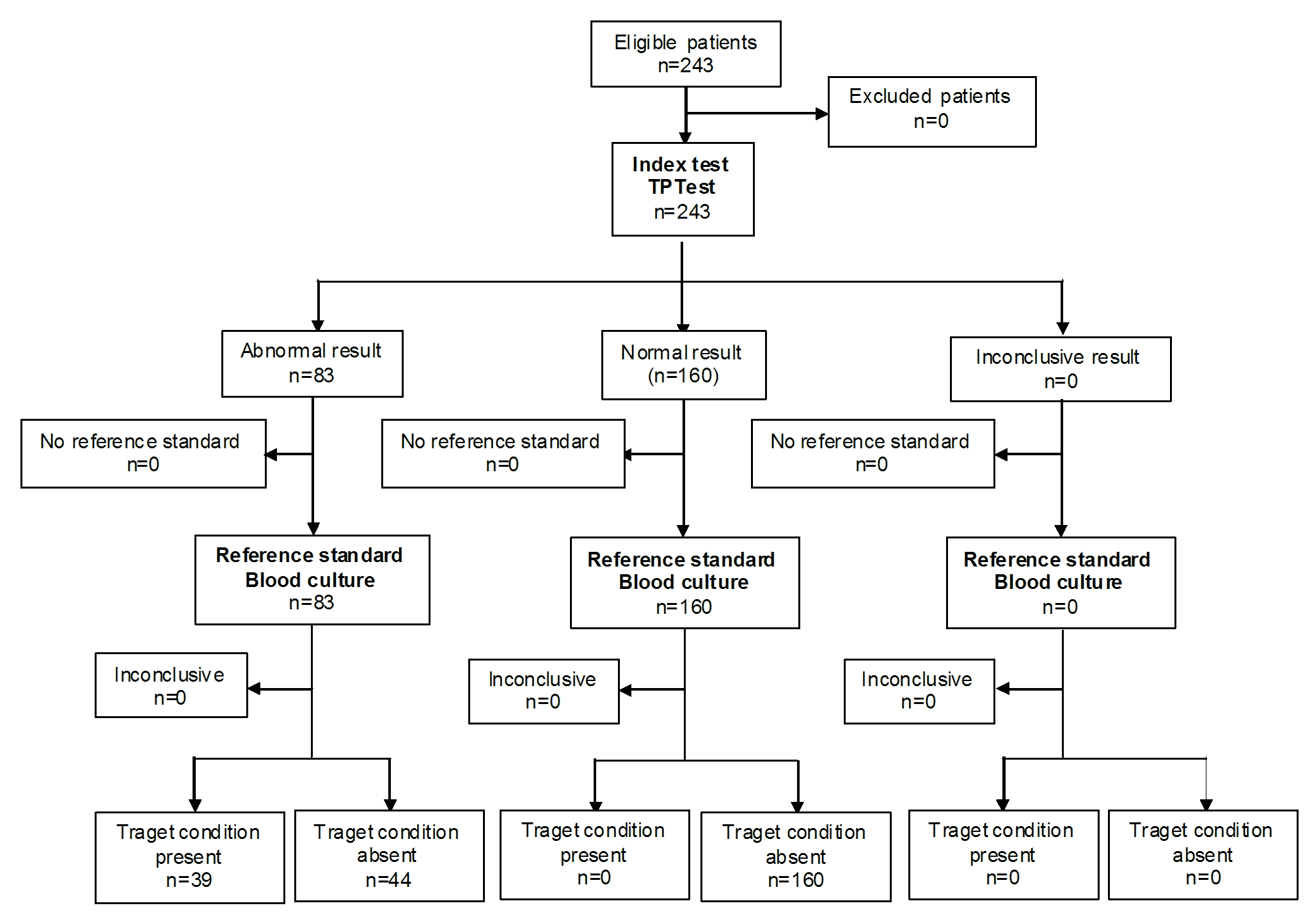

Supplement: Figure S1 — STARD flowchart of the study. (TIF) [file pntd.0002316.s001.tif]
